# Demographic reconstruction of the Western sheep expansion from whole-genome sequences

**DOI:** 10.1093/g3journal/jkad199

**Published:** 2023-09-07

**Authors:** Pedro Morell Miranda, André E R Soares, Torsten Günther

**Affiliations:** Human Evolution, Department of Organismal Biology, Uppsala University, SE-752 36 Uppsala, Sweden; Human Evolution, Department of Organismal Biology, Uppsala University, SE-752 36 Uppsala, Sweden; National Bioinformatics Infrastructure Sweden, Science for Life Laboratory, Department of Medical Biochemistry and Microbiology, Uppsala University, SE-752 37 Uppsala, Sweden; Human Evolution, Department of Organismal Biology, Uppsala University, SE-752 36 Uppsala, Sweden

**Keywords:** sheep, demography, whole-genome sequencing, gene flow

## Abstract

As one of the earliest livestock, sheep (*Ovis aries*) were domesticated in the Fertile Crescent about 12,000–10,000 years ago and have a nearly worldwide distribution today. Most of our knowledge about the timing of their expansions stems from archaeological data but it is unclear how the genetic diversity of modern sheep fits with these dates. We used whole-genome sequencing data of 63 domestic breeds and their wild relatives, the Asiatic mouflon (*O. gmelini*, previously known as *O. orientalis*), to explore the demographic history of sheep. On the global scale, our analysis revealed geographic structuring among breeds with unidirectional recent gene flow from domestics into Asiatic mouflons. We then selected 4 representative breeds from Spain, Morocco, the United Kingdom, and Iran to build a comprehensive demographic model of the Western sheep expansion. We inferred a single domestication event around 11,000 years ago. The subsequent westward expansion is dated to approximately 7,000 years ago, later than the original Neolithic expansion of sheep and slightly predating the Secondary Product Revolution associated with wooly sheep. We see some signals of recent gene flow from an ancestral population into Southern European breeds which could reflect admixture with feral European mouflon. Furthermore, our results indicate that many breeds experienced a reduction of their effective population size during the last centuries, probably associated with modern breed development. Our study provides insights into the complex demographic history of Western Eurasian sheep, highlighting interactions between breeds and their wild counterparts.

## Teaser

Sheep have been a popular livestock species ever since their domestication and have accompanied humans all over the world. Here, Miranda et al. provide a comprehensive analysis of their demographic history with a particular focus on their Western Eurasian expansion. The authors find that Western sheep descended from a single population that separated from wild mouflons during the Neolithic, and that there was an important secondary expansion, probably associated with the expansion of wool industries.

## Introduction

Sheep (*Ovis aries*) represent one of the earliest known livestock species to be domesticated in the Fertile Crescent about 12,000–10,000 years ago (ya) (Arbuckle *et al.* 2009; Stiner *et al.* 2014; Abell *et al.* 2019; Larsen *et al.* 2019) and they have been a key resource for pastoral and agricultural communities ever since. Several studies have revealed aspects of sheep history and evolution that point to a complex demographic history with an uncertain origin and multiple waves of expansion from the Middle East/Central Asia into Europe. The presence of multiple distinct mitochondrial DNA haplogroups in modern sheep that were already present before domestication (Pedrosa *et al.* 2005) has been suggested as evidence of multiple domestication events. However, a large and heterogeneous wild population, major gene flow from wild sheep into domestic flocks, or a combination of both could also explain this pattern. Studies on Y chromosomes (Deng *et al.* 2020) and polymorphic endogenous retroviruses (Chessa *et al.* 2009) have suggested the possibility of important gene flow events after the initial expansion related to the development of wool industries.

Analyses of genome-wide datasets have been successful in providing important insights into the evolutionary history of sheep breeds across the globe. Studies using single nucleotide polymorphisms (SNP) arrays showed that the species exhibits general geographic clines that match the expansions into the different regions following the Neolithic transition (Kijas *et al.* 2012; Barbato *et al.* 2017; Ciani *et al.* 2020). Contributions from diverse wild stock are also supported by the higher genetic diversity and haplotype sharing compared with other domesticates observed in modern breeds (Kijas *et al.* 2012). At the same time, population structure and demographic history on a more regional scale are consistent with a scenario in which admixture with other sheep breeds and wild ovids is more common than previously thought (Barbato *et al.* 2017; Hu *et al.* 2019; Ciani *et al.* 2020; Rochus *et al.* 2020) and may even have been encouraged in some cases to acquire desired traits from local wild ovids (Cao *et al.* 2021; Lv *et al.* 2022; Cheng *et al.* 2023). More recently, whole-genome sequencing has been employed for understanding global population structure and history (Deng *et al.* 2020; Chen *et al.* 2021; Lv *et al.* 2022), introgression from wild relatives (Chen *et al.* 2021; Lv *et al.* 2022; Cheng *et al.* 2023), how early artificial selection shaped domestic groups (Naval-Sanchez *et al.* 2018; Li *et al.* 2020), and patterns shared between sheep and other domesticated species (Alberto *et al.* 2018). While these studies highlighted the power of whole-genome sequencing data for insights into the past of this important livestock species, they were mostly focused on global patterns without addressing intra-continental patterns, aggregating together different breeds at the continental scale or by phenotypic characteristics (e.g. the presence of a fat or thin tail), which can lead to the miss-representation of continental or regional demographic patterns.

Today, sheep are a very popular livestock species along the Atlantic coast from North-Western Africa to the European Islands of the North Atlantic with hundreds of recognized breeds ranging from local landraces to popular industrial breeds. After their initial westward introduction into Europe, sheep have experienced at least one additional expansion from Western Asia, possibly associated with the development of wool industries (Chessa *et al.* 2009; Deng *et al.* 2020). In contrast to other parts of Eurasia, the absence of wild ovids in Europe facilitated feralization which later enabled back-admixture from long-term feral European mouflon into managed breeds (Barbato *et al.* 2017; Chen *et al.* 2021; Cheng *et al.* 2023). Europe was also the place where selective breeding as scientific practice started during the British Agricultural Revolution in the 18th century (Wood 1973). More recently, popular industrial breeds, such as Merino originating from Iberia, have been exported to other continents and were used for cross-breeding with many other breeds (Ciani *et al.* 2015; Ceccobelli *et al.* 2023), and some authors have hypothesized this process has been recurrent during the last millennia (Ryder 1987; Kandoussi *et al.* 2022). Altogether, this paints a picture of a complex demographic history of European sheep with a lot of uncertainties about the exact timing of particular events such as the separation of different streams of ancestry or the presence and extent of gene flow post separation of populations and breeds. Whole-genome resequencing data together with state-of-the-art statistical modeling approaches, however, should have the power to investigate these open questions.

The aim of this study is to infer the demographic history of Western Eurasian sheep from an extensive dataset of whole-genome sequences, including both landraces and improved breeds as well as wild Asiatic mouflons (*O. gmelini*). We first performed an exploratory analysis on a global panel of sheep and mouflon in order to select five representative sheep populations and propose different demographic models for the history of Western sheep. These models differ in their general topology and some of them include admixture events. We estimate split dates as well as the extent of gene flow using the site frequency spectrum (SFS) (Kamm *et al.* 2020) and investigate how their effective population sizes changed over the last millennia and centuries. This approach allows us to describe the relationship between sheep breeds in Western Europe and how they were shaped by the demographic events of the past.

## Materials and methods

### Data collection and processing

This study uses publicly available whole genomes from commercial and traditional domestic sheep breeds from the International Sheep Genome Consortium (ISGC) (Daetwyler *et al.* 2019; Kijas *et al.* 2013) and a set of wild Asiatic mouflons (*O. gmelini*) from the *NEXTGEN* project (Alberto *et al.* 2018). These datasets include 63 domestic sheep breeds from 21 countries and 935 individuals, and 18 wild mouflons from Iran (Supplementary Table 1). In addition to whole-genome data, we gathered a set of 79 mitochondrial genomes from domestic sheep, urial (*O. vignei*), argali (*O. ammon*), snow sheep (*O. nivicola*), and bighorn sheep (*O. canadensis*), publicly available in GeneBank, which were then combined with the mitochondrial genomes from the wild Asiatic mouflons and 2 ancient samples (Supplementary Table 4) from the Anatolian Neolithic, dated to 7031–6687 cal BCE and 6469–6361 cal BCE (Yurtman *et al.* 2021).

The 18 Asiatic mouflon genomes in FASTQ format were processed following the ISGC pipeline (Kijas *et al.* 2013) to keep consistency with the already processed ISCG v2 dataset. FASTQ files were mapped to the Oar3.1 reference genome using *BWA mem* (v0.7.12) (Li 2013) with default parameters, and duplicates were removed with *SAMtools* (v1.3) *rmdup* (Li *et al.* 2009). Local indel realignment was performed using *GATK* (v3.4.46) *RealignerTargetCreator* (Van der Auwera *et al.* 2013). Variant calling was performed independently using *SAMtools mpileup* and *GATK UnifiedGenotyper* with default parameters, and the resulting VCF files were filtered to remove multiallelic variants, variants never observed on one of the strands, low quality (PHRED score <20) and low mapping quality variants (PHRED score <30), variants where coverage was <10× across all samples and in cases where there were 2 variants closer than 3 bp apart, the one with the lowest quality was removed. Similarly, in the case of indels that were closer than 10 bps, the lowest quality one was removed, and variants within 5 bp of an indel were filtered. Then, an intersect of both files was created to produce the final VCF file using *GATK CombineVariants* and *SelectVariants*.

To avoid issues with over-representation of some breeds, the dataset was subsampled to, at most, 5 randomly selected samples per breed, and only SNPs with a minor allele frequency (MAF) of 0.05 and a genotyping rate of 0.9. These filters resulted in a dataset of 176 samples (Supplementary Table 2) and 16932388 SNPs.

Mitochondrial consensus sequences were called from FASTQ sequences with *MIA* (Green *et al.* 2009), a reference-based iterative assembler, using the reference sequence for the Asiatic mouflon (NCBI Reference Sequence NC_026063). To ensure reliability on the bases called, a minimum of 10 unique molecules (10× coverage) was necessary for a consensus to be called for each position, plus a minimum map quality of 40 and two-thirds base agreement on each position. Any site that did not meet any of these quality parameters was called “N.” All sequences were then manually assessed and aligned using *MAFFT* (v7.407) (Katoh and Standley 2013).

### Exploratory population genetic analysis

In order to explore the relationship between the different breeds, we performed a principal component analysis (PCA) using *SmartPCA*, from the *EigenSoft* (v7.2.1) package (Patterson *et al.* 2006; Price *et al.* 2006). To avoid the effect of linkage disequilibrium (LD), data was pruned using *Plink* (v1.90b4.9)’s parameter --indep option (5001.066) (Purcell *et al.* 2007). Further population structure analysis was performed using *ADMIXTURE* (v1.3) (Alexander and Lange 2011). The number of assumed clusters ranged from 2 to 10, and each cluster was run with different random seeds 20 times. The results were compared and plotted using *Pong* (v1.4.9) (Behr *et al.* 2016).

**Fig. 1.**
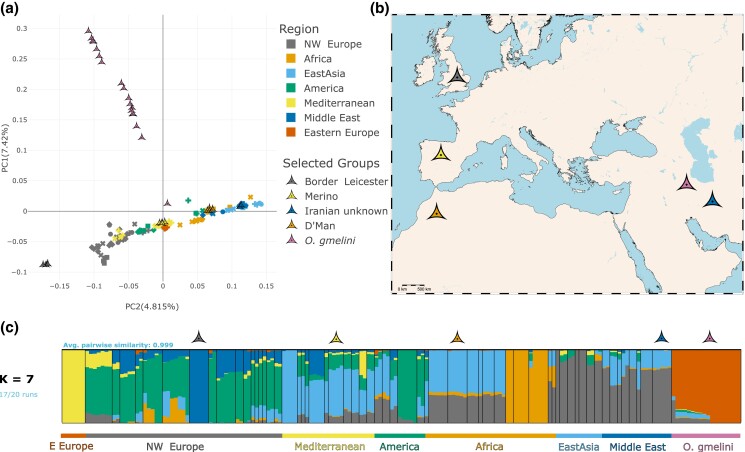
a) Graphic representation of the first 2 principal components (PCs) of a principal component analysis (PCA) calculated using 63 domestic breeds and Asiatic mouflons. PC1 describes the variation within wild mouflons, with some individuals showing signs of admixture with domestics sheep. PC2 captures the variation within domestic sheep and presents a geographical pattern, with European breeds on the negative extreme, and East Asian breeds on the positive. b) Geographical origin of the 5 breeds used for demographic inference. Map created using pyGTM (Tian *et al*. 2023). c) Proportion of each sample’s genome assigned to K=7 genetic clusters using *ADMIXTURE*. Wild mouflon ancestry is almost exclusively observed in wild samples, while Eastern Eurasian samples present mostly a single ancestry cluster. Middle Eastern and African samples show variable levels of ancestry from Eastern and Mediterranean ancestry, and Mediterranean and North-Western breeds show high levels of admixture from each other and Eastern European.

Data was then subsampled to a set of 5 representative populations: Border Leicester and Merino as proxy for North-Western and South-Western European sheep, respectively, D’Man for North African sheep, and a group of Iranian sheep of no formally described breed, in the original data labeled as “Unknown,” for Middle Eastern sheep (Fig. 1b, Supplementary Table 3). To explore the splits and admixture events between our groups, we created admixture graphs with *OrientAGraph* (v1.0) (Molloy *et al.* 2021) using the LD pruned dataset and the *-mlno -allmigs* parameters. To assess the robustness of the results and avoid local optima, *OrientAGraph* was run using the *-bootstrap -k 500* option to create 10 replicates. All trees were visually inspected using the *Treemix* (Pickrell and Pritchard 2012) plotting functions on *R* (v4.2.2) (R Core Team 2022) and the one with the highest likelihood was then selected.

### Demographic modeling

Informed by the results of the exploratory analysis, we defined 5 models for the demographic history of Western sheep to be tested with *Momi2* (v2.1.16) (Kamm *et al.* 2020). The models were defined as follows:

- **Model A:** Single domestication event without admixture, assuming Iranian and Moroccan sheep form a monophyletic group.
- **Model B:** Single domestication event assuming Moroccan sheep are a sister group to European sheep. A variant of this model with admixture from wild mouflons into Merino, as suggested by *OrientAGraph* with 1 migration edge, was also considered.
- **Model C:** Single domestication event assuming Moroccan sheep are a sister group to European sheep with an admixture event into the Merino sheep, as suggested by *OrientAGraph* with 1 migration edge. In contrast to the gene flow variant of Model B, the source of the gene flow originates from an ancestral domestic ghost population and not the wild mouflon branch.
- **Model D:** Two independent domestication events from different regions inside of the Fertile Crescent for Eastern and European Sheep. A variant of this model with admixture from wild mouflons into Merino, as suggested by *OrientAGraph* with 1 migration edge, was also considered.
- **Model E:** Two independent domestication events from different regions inside of the Fertile Crescent for Eastern and European Sheep with admixture into Merino sheep, as suggested by *OrientAGraph* with 1 migration edge. In contrast to the gene flow variant of Model D, the source of the gene flow originates from an ancestral domestic ghost population and not the wild mouflon branch.

**Fig. 2.**
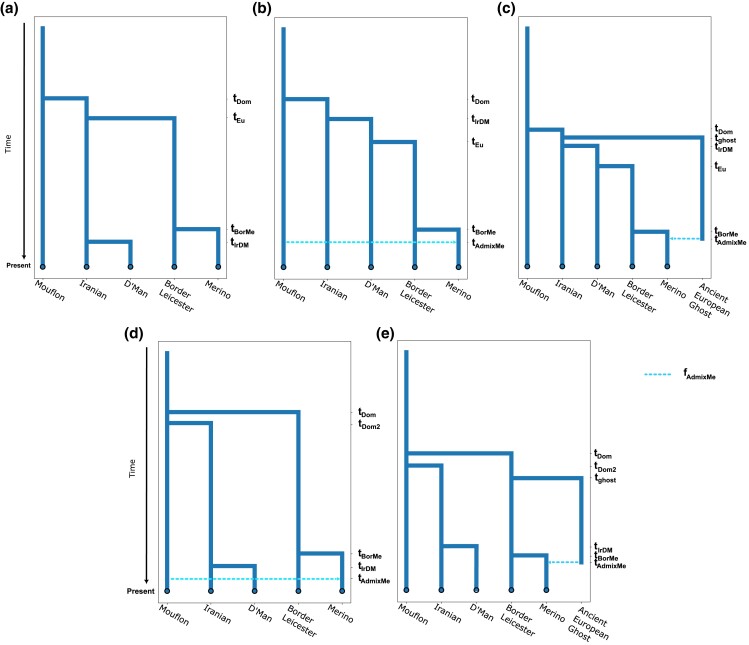
Topologies for the demographic models tested, displayed here with 1 admixture pulse (if admixture was considered). a) Single domestication event with Moroccan and Iranian sheep as a monophyletic group. b) Single domestication event assuming Moroccan sheep are a sister group to European breeds with an admixture event from wild relatives. c) Single domestication event assuming Moroccan sheep are a sister group to European breeds with an admixture event from a domestic ghost population. d) Independent domestication of Eastern and Western sheep with gene flow from wild mouflons into Merino. e) Independent domestication of Eastern and Western with admixture from a domestic ghost population. The dashed lines represent an admixture pulse.

A schematic representation of all model topologies is shown in Fig. 2. All branches in the models were allowed to have a different population size and all domestic breeds were allowed to grow freely, split times were only constrained by the order of splits defined by the model topology and with a starting value based on prior information (e.g. for the domestication time, all models were set to start at 12,000 ya, see Supplementary Table 7 for a detailed description). As the curated dataset with MAF filters and subsampling of individuals likely removed most novel mutations in the time since domestication, *Momi2* was set to internally estimate mutation rate from the data while a generation time of 3 years, i.e. the average parental age at reproduction, was assumed (Young and Purser 1962; Rafter *et al.* 2022). Allele frequencies and the SFS were calculated for 5 individuals of each of the 5 selected populations using *Momi2* (Kamm *et al.* 2020), the optimization of the model was done using the *L-BFGS-B* algorithm and model fit was evaluated both with logLikelihood and the Kullback–Leibler divergence parameter (Kullback and Leibler 1951), and each model’s parameters were estimated from 20 independent runs and the best-fitting model (based on their likelihood) was then chosen. Confidence intervals (CI) were calculated using the results of *Momi2* bootstrapping runs (n=100) for each parameter from the empirical extremes by using *numpy.percentile* (Harris *et al.* 2020) with a confidence of 95%.

### Effective population size estimation

To investigate the effective population size ($N_{e}$), we combined the results of 2 methods of $N_{e}$ inference that complement each other in the time-span covered (Nadachowska-Brzyska *et al.* 2022): *SMC++* (Terhorst *et al.* 2016) for deep-time $N_{e}$ estimations and *GONE* (Santiago *et al.* 2020) for more recent changes. Data from all available samples from each population was used. *SMC++* input data was prepared using the *vcf2smc* function with default settings on all unphased autosomal chromosomes, and run with *estimate* with generation time set to 3 years and mutation rates of $2.5\times 10^{-8}$, $1\times 10^{-8}$, and $5.87\times 10^{-9}$ were tested. *GONE* input data was prepared using *Plink* (v1.90b4.9) to produce *ped* and *map* files on unphased autosomal chromosomes and was run with standard settings except for a MAF filter of 0.1 and a generation time of 3 years. To validate the observed patterns in GONE, we detected runs of homozygosity (ROHs) using *Plink* (v1.90b4.9)’s *—homozyg* function (Purcell *et al.* 2007) with a maximum inverse density of 50, a maximum gap of 1,000, a window threshold of 0.02, a window size of 100 SNPs, max missingness of 10, allowing for only 1 heterozygous SNP per hit and with a minimum length of 50 kb.

### Mitochondrial phylogeny

A phylogenetic analysis using mitochondrial data was performed. All sequences were aligned using *MAFFT* (v7.407) (Katoh and Standley 2013). We then created phylogenetic trees using both a maximum likelihood (ML) and a Bayesian approach (Bayesian inference, BI). For the ML tree, we used *IQ-TREE* (v2.1.3) (Chernomor *et al.* 2016), and allowed the algorithm to infer the best substitution model using *Model Finder Plus*. We estimated the phylogeny and divergence time of the alignment using the Bayesian genealogical inference package *BEAST* (v2.5) (Bouckaert *et al.* 2014). We assumed the GTR+Γ nucleotide substitution model under an uncorrelated lognormal relaxed clock with a Yule process tree prior and 2 calibration points (Drummond *et al.* 2006). We used as calibration points the separation of *O. dalli* and *O. canadensis* from the Eurasian species (normal prior centered at 1.57 Mya, according to Rezaei *et al.* 2010), and the group containing *O. gmelini*, *O. ammon*, and *O. vignei* (normal prior centered at 1.72 Mya (Rezaei *et al.* 2010]). We also used tip dating calibration for both Anatolian Neolithic samples (*tps062* and *tps083*) according to radiocarbon dates reported in Yurtman *et al.* (2021). We combined 3 independent Markov chain Monte Carlo (MCMC) runs to ensure proper mixing of the chain. Each chain ran for 100 million iterations, discarding the first 20% as burn-in. We visualized convergence of the MCMC chains by eye using *Tracer* (v1.6) (Rambaut *et al.* 2018) and calculated the maximum clade credibility tree using *TreeAnnotator* (v2.5) (Suchard *et al.* 2018). The final tree was edited with *FigTree* (v1.4.3) (Rambaut 2016).

## Results

The main goal of this study was to reconstruct the demographic history of the Western sheep expansion. The enormous number of possible models does not allow us to perform explicit demographic modeling for all possible breeds together and we need to restrict the model space by reducing the number of populations. Therefore, we initially performed an exploratory analysis of publicly available genome data from a worldwide dataset of sheep breeds and wild Asiatic mouflon (Alberto *et al.* 2018; Daetwyler *et al.* 2019). Based on the results of this analysis, we selected representative breeds for a model-based reconstruction of their demographic history from whole-genome sequences which is complemented by a phylogenetic reconstruction of the maternally inherited mitochondrial genome.

### Global population structure

To obtain a general overview of the relationship between sheep breeds of different regions of the world, we performed a PCA of the genome-wide variation. The first major axis of variation (PC1) separates wild mouflon from domestic sheep breeds (Fig. 1a). Mouflons are spread along this axis, which may suggest some heterogeneity within this group with regard to the relationship to sheep breeds and/or gene flow between domesticated sheep and wild mouflon (Ciani *et al.* 2020; Deng *et al.* 2020; Luigi-Sierra *et al.* 2020). PC2 shows a clear distinction between different domestic sheep groups, which exhibit a geographic pattern across Eurasia with North-Western European sheep showing more positive values while Eastern Eurasian breeds tend to have negative values, and Mediterranean, Middle Eastern and African breeds are distributed along this East-West gradient. Breeds with a known history of admixture such as Romanov and Dorper sheep fall between the 2 big continental groups. These 2 modern breeds are known to carry mixed Eastern and Western ancestry and were bred to adapt to the harsh climate conditions in Russia and South Africa, respectively (Milne 2000; Deniskova *et al.* 2018).

The genomic clustering analysis with *ADMIXTURE* (Alexander and Lange 2011) presented a similar pattern (Supplementary Fig. 1) as the PCA, with K=2 discriminating between Asiatic mouflon and domestic sheep while some mouflons show signals of domestic sheep admixture and a small proportion of mouflon ancestry is seen in several domestic sheep. The latter signal, however, disappears for K=3 and above (Supplementary Fig. 1) where we observe a split between Eastern and Western sheep clusters with most commercial Western breeds heavily admixed. At K=7 (Fig. 1c), we see a complex pattern of regional ancestries, with Eastern Asian breeds showing low levels of cluster diversity, while Middle Eastern and African breeds show a higher component in common with Mediterranean sheep, and European breeds seem to be a mix of 3 clusters: 1 more common in Mediterranean and North African breeds and 2 mostly present in North-Western European breeds, plus some gene flow from Eastern Europe and Eastern Asia. Higher values of *K* produce various different modes according to the *pong* (Behr *et al.* 2016) analysis, indicating multiple local optima (Supplementary Fig. 1). Across all *K*s, some Asiatic mouflons show admixture from domestic sheep, while gene flow from wild mouflons into domestic sheep (including the sympatric Iranian sheep) appears to be minor or nonexistent.

Gene flow from domestic species into mouflons is also supported by the mitochondrial phylogeny inference (Supplementary Figs. 5 and 6). Both trees show 1 mouflon clustering within the A haplogroup, with the most recent split being the Anatolian Neolithic sheep *tps083*. All other mouflons except one which seems to have been admixed with urials (*O. vignei*), belong to the CE haplogroup complex, but 3 mouflons also fall within a domestic branch, supporting the idea of domestic introgression from different sources into this wild population.

Based on this exploratory analysis, we selected the wild mouflons as an outgroup and 4 domestic sheep populations covering different extremes of the Western sheep expansion for subsequent analysis (Fig. 1b). The selected domestic populations were Border Leicester (United Kingdom), Horned Merino (Spain), D’Man (Morocco) and a set of traditional Iranian sheep that have not been properly defined into a breed, which are labeled as “Unknown” in the original data. These breeds were selected to serve as proxies for the origin and the extremes of the Western expansion of sheep from the Fertile Crescent, they had a sufficient number of samples for further analysis (>19), and since both PCA and *ADMIXTURE* show them as independent populations from each other. More comprehensive analyses of the global population structure of sheep have been previously published (Kijas *et al.* 2012; Naval-Sanchez *et al.* 2018; Ciani *et al.* 2020; Li *et al.* 2020; Lv *et al.* 2022).

### Structure and gene flow in Western Eurasian sheep

**Fig. 3.**
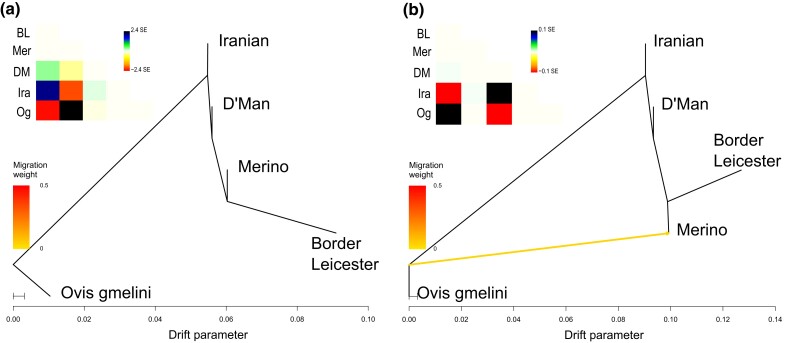
Maximum Likelihood Network Orientation reconstruction for the relationship between 4 domestic breeds and the Asiatic mouflon using *OrientAGraph*. a) The tree without migration edges displays a pectinate topology with the Iranian sheep splitting first, and D’Man being a sister group to both European breeds. The residual plot show high values for the intersection between wild mouflons and Merinos and between Iranian sheep and Border Leicester. These are resolved adding b) 1 migration edge from the base of the tree into Merino. The columns of the residual matrices follows the same order as the rows.

Using the representative subset of breeds as outlined above, we moved towards a more comprehensive analysis of their relationship. We used *OrientAGraph* (Molloy *et al.* 2021) to explore possible topologies of models to test. The initial tree (without migration edges) follows a pectinate topology with Asiatic mouflon as an outgroup to domestic sheep then Iranian “Unknown” splitting off followed by D’man and finally Border Leicester and Merino as sister groups (Fig. 3a). The residual covariance suggests some shared, unexplained ancestry between Asiatic mouflon and Merino which is resolved by a migration edge from the root of the tree into Merino (migration weight 0.0379) once 1 migration event is allowed (Fig. 3b). At this point, most of the allele frequency covariance matrix is explained by the model with residuals only around ±0.1 standard errors (SE). Therefore, we decided to restrict our models for the more explicit demographic reconstruction to models with a maximum of 1 gene flow event.

### Demographic reconstruction

**Fig. 4.**
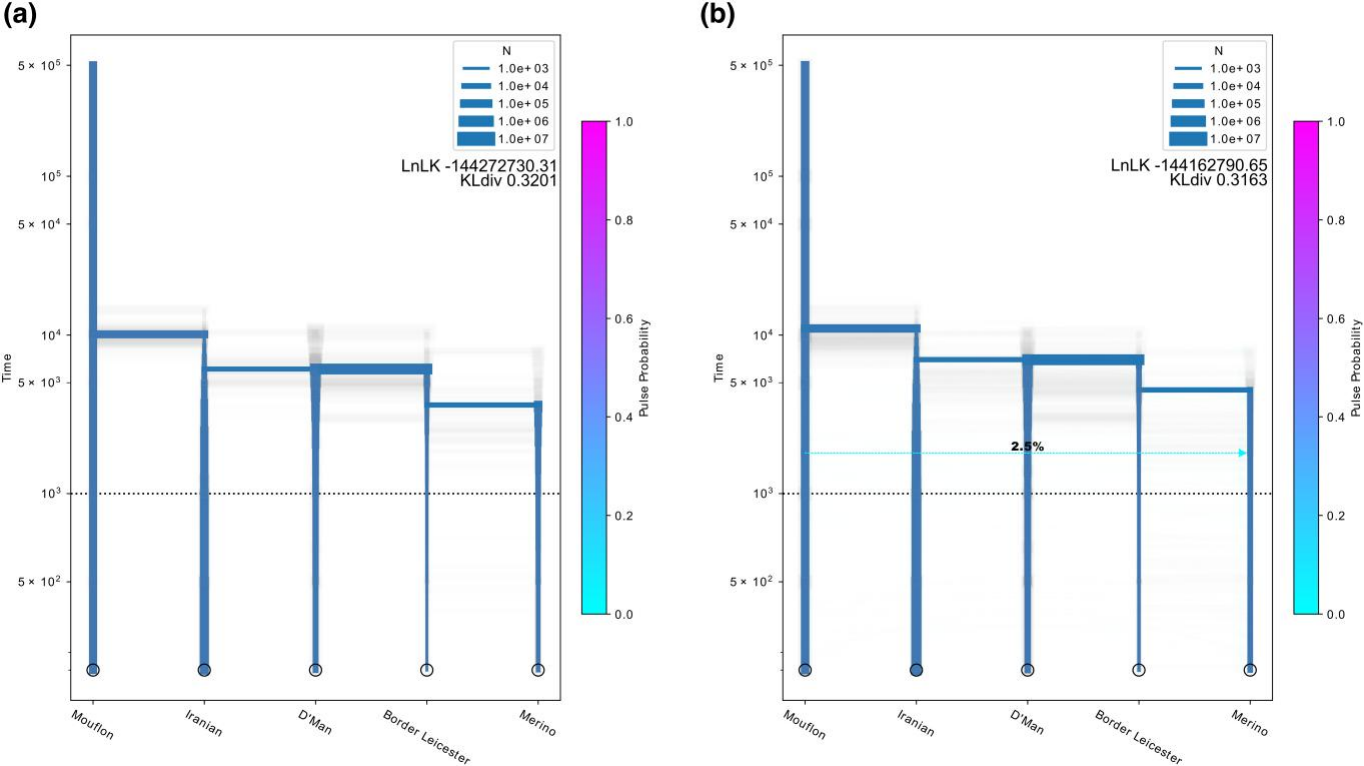
Maximum likelihood inference of the demographic history of Western sheep using *Momi2*. a) Model B without admixture shows the same topology as *OrientAGraph* without migration pulses, with narrow confidence intervals around the expected time of domestication. b) Model B with admixture, which displays the same topology as Model B, but adds admixture from a wild population. The model shows similarly narrow split times close to the expected domestication period. Time is in years assuming a generation time of 3 years. The width of the branches indicates effective population sizes.

Following these results and scenarios based on the literature (Pedrosa *et al.* 2005; Barbato *et al.* 2017; Ciani *et al.* 2020), we defined 5 demographic models in order to reconstruct the demographic history of Western sheep (Fig. 4). We used the SFS inferred from the whole-genome sequences of the 5 populations to test these models using *Momi2* (Fig. 4, Supplementary Fig. 2 and Table 1, Supplementary Tables 5 and 6) (Kamm *et al.* 2020). The best-fitting model was model B with gene flow (logLikelihood: −144162790.65, Kullback–Leibler divergence, or KLdiv, between the predicted and observed SFS: 0.3163268), which assumes a single domestication event and that North African sheep are a sister group to European sheep and admixture from wild mouflons into Merino. Closely behind was Model B (logLikelihood: −144272730.31, KLdiv: 0.3200644), which describes the same topology, but with no admixture. As the difference is quite marginal and Model B has less parameters (Table 1) and a denser distribution of bootstrapping estimations around the best model values, we display both variants in Supplementary Fig. 2. Model C, which describes the same demography but with gene flow from a basal domestic ghost population, fits the data slightly less well (logLikelihood: −144286373.27, KLdiv: 0.3205282). Models that assumed a double domestication were the ones that performed worst (Table 1). Lastly, model A, which describes African sheep as a sister group to Iranian sheep, performs at a similar level to models B and B with admixture in 1 of the 20 runs (logLikelihood: −144268904.28, KLdiv: 0.3199343), but the bootstrapping values show quite wide distributions on all events and several local minima, while the other models are quite less spread (Supplementary Fig. 2). Model A is also an outlier in that time estimates are far more recent than any other model (Supplementary Table 6), so recent that they are unrealistic with the wild-domestic split substantially postdating archaeological dates for domestication.

**Table 1.** Performance of the different models tested by *Momi2*.

Model	LogLikelihood	KL divergence	Parameters
A	−144268904.28	0.3199343	13
B	−144272730.31	0.3200644	13
B with gene flow	−144162790.65	0.3163268	15
C	−144286373.27	0.3205282	16
D	−150986849.27	0.5483216	13
D with gene flow	−149644568.67	0.5026886	15
E	−149612640.95	0.5016032	16

Estimated values for split times in the 2 best models roughly agree with archaeological evidence for the date of domestication (Zeder 2008; Stiner *et al.* 2022). Model B estimates that the split between wild mouflons and domestic sheep happened 10,114 ya (95% CI [7,785, 14,500]). For Model B with admixture a very similar split time is estimated at 11,007 years in the past (95% CI [6,769, 14,459]). The two models show that the split between Iranian and Western sheep and North African and European sheep happened in quick succession (6,114 and 6,112, respectively, for Model B, and 7,007 and 6,996 for Model B with admixture) (95% CIs [3,785, 10,500]; [2,948, 10,493]; [2,769, 10,459] and [2,768, 10,163]) (Supplementary Table 6) but after the initial expansion of this species westwards during the Neolithic. Both models, however, disagree on the timing of the European split, with Model B estimating it at 3,613 ya (95% CI [448,7,993]) and Model B with admixture at 4,497 ya (95% CI [269,7,664]). This final split is also where both models’ bootstrapping values show an extreme spread (Fig. 4), which seems to be in connection with the admixture pulse in Model B with admixture, estimated to have happened at 1,802 ya with a weight of 0.025 (Fig. 4). These estimates also have quite wide CIs, ranging from 32 to 6,757 ya and a weight between 0.000045 and 0.9431.

In terms of effective population size of our 4 domestic breeds, both *Momi2* models show a steep bottleneck in the population ancestral to all domestic breeds (10,114 and 11,007 ya, respectively), as expected for this kind of process (Fig. 4). The Iranian branch, however, quickly starts to grow in both models. Both models also agree that the ancestral population of the two European breeds started shrinking shortly after the split from D’Man. The effective population size in Border Leicester continues to decrease while the size of the Merino branch is small but relatively steady. They also agree on the Moroccan population, displaying a bigger $N_{e}$ after the split, that slowly decreases and stabilizes before 1,000 ya.

### Effective population size over time

**Fig. 5.**
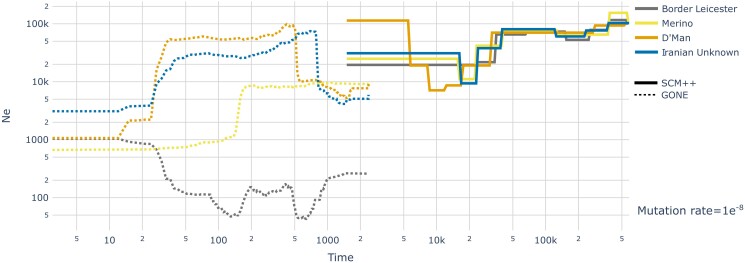
Effective population size inference of the 4 domestic sheep populations used in the demographic history reconstruction using *SMC++* and *GONE*. *SMC++* describes an ancient bottleneck around 150,000–400,000 ya, followed by a more recent, stronger bottleneck starting around 40,000 ya with most breeds reaching a minimum around 20,000 ya (i.e. slightly before the expected domestication period) and a subsequent increase in $N_{e}$ that is independent for each population. *GONE* describes more recent changes in $N_{e}$. D’Man and the Iranian unknown sheep seem to have experienced an increase of $N_{e}$ around 700 and 500 ya, respectively, and then a bottleneck, while Merinos show a strong bottleneck 150 ya. Border Leicester seems to have undergone several strong bottlenecks recently with a fairly recent rise in $N_{e}$. Time is in years assuming a generation time of 3 years and a mutation rate of $1\times 10^{-8}$.

The *Momi2* results indicate a reduction of population size in each individual breed after they split from the other populations. To obtain a more detailed picture on this development with temporal resolution, we employed a combination of *SMC++* for the last millennia (Terhorst *et al.* 2016) and *GONE* for the last centuries (Santiago *et al.* 2020) (Fig. 5) to try to cover both deep and recent $N_{e}$ changes (Nadachowska-Brzyska *et al.* 2022). Due to the lack of direct estimates of the nuclear mutation rate of sheep, we used 3 different values, 2 used in previous studies ($1\times 10^{-8}$ and $2.5\times 10^{-8}$, Alberto *et al.* 2018; Hu *et al.* 2019; Li *et al.* 2020; Chen *et al.* 2021; Cheng *et al.* 2023; Zhu *et al.* 2023) and 1 direct estimate for goats ($5.87\times 10^{-9}$, Bergeron *et al.* 2023) (Supplementary Fig. 3). All 3 runs of *SMC++* display similar curve shapes, with an ancient bottleneck and a more recent, severe one, but differ substantially on the time scale. Using the empirical mutation rate for goat, $5.87\times 10^{-9}$, the recent bottleneck for all domestic sheep begins before 50,000 ya, while the highest mutation rate, $2.5\times 10^{-8}$, places the start of the bottleneck at slightly before the 20,000 ya mark. Using $1\times 10^{-8}$, *SMC++* shows the recent bottleneck beginning around 40,000 ya. After this supposed domestication bottleneck, each population increases in size at different times and rates. We display the results for the intermediate mutation rate, $1\times 10^{-8}$, in Fig. 5 and the other mutation rates in Supplementary Fig. 3.

*GONE* estimates are considered most reliable in more recent periods (Santiago *et al.* 2020). Indeed, we see that the *GONE’*s $N_{e}$ estimates for about 300–500 ya are similar to the most recent estimates from *SMC++* from about 1,500 ya for all breeds except Border Leicester, which has a much lower population size according to *GONE* compared to *SMC++*. The other breeds show a reduction of their effective population size during the last centuries which could be associated with the appearance of modern breeding practices (Ryder 1964) and, in the case of Merino, the fact that the sequenced samples had been introduced to Australia. Border Leicester show an almost mirrored trajectory with extremely low effective population sizes up to the last century followed by an increase to similar levels as the other breeds. This is validated by a different length distribution of Runs of Homozygosity between the 4 breeds (Supplementary Fig. 4). Border Leicester has the most shortest homozygous segments, while the other breeds tend to have fewer but longer segments. This suggests more historical inbreeding in Border Leicester, while the other breeds have seen an increase of inbreeding in recent times, consistent with the *GONE* results (Fig. 5). Border Leicester derives from Dishley Leicester, one of the first breeds subjected to selective breeding as a scientific practice (Young and Purser 1962) making it possible that the bottleneck associated with modern breeding practices started earlier and/or was more aggressive for these sheep. The recent recovery of effective population size could reflect the current popularity of the breed in Great Britain and other regions of the world or could correspond to cross-breeding with other stocks, although it is disputed whether Border Leicester were kept purebred during the last centuries (Young and Purser 1962). In both methods, the recent as well as most of the historical effective population size is similar or lower in the two commercial European breeds compared to D’man and the Iranian sheep. Our estimates of the current effective population size quantitatively differ from some other breed-specific estimates in the literature (e.g. Kijas *et al.* 2012) which likely reflects different methodologies and their sensitivity to recent $N_{e}$ changes as our *GONE* results suggest strong differences between $N_{e}$ trajectories in the last century.

## Discussion

### Structure of modern sheep populations and gene flow

Our analysis corroborated the known geographical structure of domestic sheep populations (Kijas *et al.* 2012; Ciani *et al.* 2020; Taylor *et al.* 2021; Yurtman *et al.* 2021). This geographical pattern is consistent with all current hypotheses about the origin and following expansion of sheep. *ADMIXTURE* results (see Fig. 1c) suggest that admixture between domestic sheep breeds has been common, especially in Europe but also in Africa and the Middle East, where both commercial and traditional breeds display a variety of ancestry components both from Europe and other regions of Eurasia. In traditional breeds, these seem to mainly receive admixture from geographically linked regions (e.g. Northern African populations being a mix of Mediterranean and Middle Eastern ancestries), with only minor contributions of really distant populations (e.g. small components of Northern European and Eastern European components in Eastern Asian breeds). In Europe, however, admixture between European breeds has been common, and Mediterranean breeds also show a significant amount of admixture with Eastern breeds. This pattern may be explained by constant trade and population movements during historic times, plus the advent of modern breeding practices in Europe and their expansion in later colonial times.

While we see this widespread gene flow between domestic groups, there is little evidence to support the hypothesis of major admixture of domestic sheep breeds with Asiatic mouflons. The only populations that show some level of wild mouflon ancestry are some European breeds, matching previously published results (Alberto *et al.* 2018) (Fig. 1). An alternative source for this signal is admixture with a population related to ancestral domestic sheep, as suggested by *OrientAGraph* (Fig. 3). We propose that this archaic population is the European mouflon, assumed to represent a feral Neolithic sheep lineage from the islands of Sardinia and Corsica. Previous studies using low-density SNP genotyping suggested low levels of European mouflon admixture into Iberian sheep breeds (Barbato *et al.* 2017; Ciani *et al.* 2020). Our best-fitting model in *Momi2* is consistent with this interpretation as the timing of the admixture event into Merino ancestors would be extremely unlikely from an Asiatic mouflon. In contrast, gene flow in the opposite direction, from domestic sheep into wild Asiatic mouflon, appears to have taken place more frequently (Fig. 1a and c, Supplementary Figs. 5 and 6).

### Demographic modeling and split dates

Our demographic reconstruction shows little to no support for the multiple domestication hypothesis. *OrientAGraph* and *Momi2* support a single domestication event with a pectinate topology (Figs. 3 and 4). In fact, when we fitted the double domestication models to our data, the estimated dates for both domestication events overlapped temporally at ∼12,000–13,000 ya (best performing model’s 95% CI [11,593, 13,900]) (Supplementary Table 6). Thus, they resemble a trifurcation soon after a single domestication event, but not necessarily 2 independent domestication events from different wild populations. We need to highlight, however, that our focus was the Western expansion of sheep and we only had sequences from an Eastern Asiatic mouflon population available (see also discussion below), which leaves possibilities for different contributions from other mouflon populations and a different history for Eastern Eurasian breeds.

While the exploratory analysis of the global dataset highlighted the abundance of recent admixture events between individual breeds, the motivation for the selection of the 5 populations used was to study deep demographic changes. Consequently, our demographic analysis points to little to no admixture between these 5 populations. Our 2 best-fitting models’ split dates between wild mouflon and domestic sheep are just slightly over ∼11,000 ya but with CIs ranging from 6,769 to 14,459 and from 7,786 to 14,500, respectively. The point estimates fall within the expected range for the domestication according with archaeological evidence (12,000–10,000 ya, Zeder 2008; Stiner *et al.* 2022). However, the sequenced modern wild mouflon population is not likely to represent the direct descendants of the wild ancestors of domestic sheep which should push the estimated split times further back in time. While the historical range of this species completely covered the putative domestication regions, its current range only marginally overlaps with it (Zeder 2008; Michel and Ghoddousi 2020) suggesting that the original source population may no longer exist in the wild. Wild mouflon populations seem stable in their total range (Michel and Ghoddousi 2020; Chen *et al.* 2021) but their populations at a local scale have decreased in the last millennia (Michel and Ghoddousi 2020; Ouhrouch *et al.* 2021), which matches with their disappearance from regions of the Fertile Crescent (Yeomans *et al.* 2017). Furthermore, the genetic variation found in domestic sheep also does not appear to be a subset of the sequenced mouflon population. This is exemplified by the mitochondrial haplogroups, Asiatic mouflons clustered with the CE haplogroup, while most Western breeds fall into haplogroups A and B. The maternal split between CE and (A, B) is even dated to about 1 million ya predating the domestication by 2 orders of magnitude consistent with other studies (Pedrosa *et al.* 2005; Meadows *et al.* 2011; Lv *et al.* 2015; Sanna *et al.* 2015; Deng *et al.* 2020).

The relatively recent estimates for the autosomal split may be explained by early gene flow from different wild populations after the initial domestication or by uncertainty regarding the appropriate generation time used to obtain these estimates. Generation time in wild sheep is estimated at around 6 years (Pacifici *et al.* 2013) due to males gaining dominance late during their life span. Domestication and herding practices have reduced this time with estimates ranging from 2 years to ∼4 years in different breeds (Young and Purser 1962; Joakimsen 2009; Tahmoorespur and Sheikhloo 2011; Stachowicz *et al.* 2018; McManus *et al.* 2019; Rafter *et al.* 2022). Therefore, the assumption of a constant generation time used in demographic reconstructions is likely violated for the comparison between wild and domestic populations where each branch might have its own generation time. A recent study used simulations and a maximum likelihood approach to infer the split time for the onset of the domestication bottleneck, which was dated to 13,609 using a generation time of 3 years (Lv *et al.* 2022). Using also a generation time of 3 years, another recent study employed approximate-Bayesian computation to date the sheep/mouflon split to about 10,500 ya (Deng *et al.* 2020), similar to our estimates and closer to the estimated domestication period based on archaeological remains. The 95% highest posterior density interval (HPDI), however, included the maximum of the prior distribution used in that analysis (12,000 years) suggesting that the point estimate of 10,500 may be an underestimation. In summary, the slightly recent estimate for the split time is likely due to a combination of the uncertainties of the different estimates and a complex domestication process that is not well modeled by a simple split event.

Another important parameter for demographic reconstructions is the mutation rate. This uncertainty manifests in the uncertainty of $N_{e}$ estimates in the *SMC++* analysis, where the time axis varies substantially depending on the mutation rate used. The only variant where the timings of the supposed domestication bottleneck matches our demographic reconstruction and expectations from archaeology uses a mutation rate of $2.5\times 10^{-8}$ mutations per generation (Supplementary Fig. 3). This value has been quite popular for demographic reconstructions in the sheep literature (Alberto *et al.* 2018; Li *et al.* 2020; Chen *et al.* 2021), but it is higher than any direct estimation on any mammal (Bergeron *et al.* 2023). Using a mutation rate of $1\times 10^{-8}$, a value falling into the range of many mammalian species (Bergeron *et al.* 2023), pushes the domestication bottleneck around 20,000–40,000 ya, while using the goat mutation rate which is the closest relative for which we have a direct estimate (Bergeron *et al.* 2023), this bottleneck ends before the Last Glacial Maximum. Therefore, biologically realistic mutation rates result in $N_{e}$ curves with timings that do not fit archaeology which can only be met using relatively high mutation rates. This highlights the need for accurate estimates of the mutation rate for each species when scaling the results of demographic reconstructions. As we do not have a direct estimate for sheep, it is advantageous that our *Momi2* modeling and the split date estimates do not depend on a priori inputs for the mutation rate, as it is estimated directly from the data.

The second split (∼7,000 ya) in our best-supported model describes almost a trifurcation between the Iranian, North African, and European branches, with only a few years of difference between population splits. This is over a thousand years after the initial Neolithic Expansion, which suggests that this part of the tree does not reflect the initial expansion of sheep after their domestication. These split times, nonetheless, predate the appearance of the Secondary Product Revolution and the presumed expansion of wooly sheep from Central Asia into the Fertile Crescent, Europe, and Northern Africa (Sherratt 1981; Marciniak 2011). As at least all the Western breeds display the wooly phenotype, this may be interpreted as most parts of the modern gene pool reflecting a secondary expansion westwards.

## Conclusions

Our results add to a mounting body of evidence on the complexity of the demographic history of sheep as well as the limitations of using genomic data from present-day populations to reconstruct it. Even for a restricted sample of five populations and a restricted geographic focus, it remains difficult to narrow down certain demographic events. Recent demographic changes in both commercial breeds and wild relatives make inferring ancient events particularly difficult, as the near-extinction of the Asiatic mouflon in a significant part of its original range or modern breeding practices could complicate demographic analysis. We were able to discriminate between several demographic models in favor of a single domestication event with domestic sheep radiating from the Fertile Crescent both eastwards and westwards. Dating these events, however, proved quite tricky. Our results point to the domestication happening from a highly diverse stock of Asiatic mouflon. This high diversity could be interpreted as evidence for multiple domestication events or, as our modeling suggests, a single domestication event in combination with introgression from diverse and significantly structured Asiatic mouflon populations, which we have not sampled or are not even present today. It is difficult to study such ancient events from the distribution of modern diversity alone and genomic ancient DNA from the domestication region could illuminate the demographic history of sheep as archaeogenomic studies have led to an enormous improvement in our understanding of domestication and subsequent gene flow in goats (Daly *et al.* 2018, 2021), pigs (Frantz *et al.* 2019), and cattle (Verdugo *et al.* 2019) already.

## Supplementary Material

jkad199_Supplementary_Data

## Data Availability

Data from the Sheep Genome Consortium v2 dataset can be accessed through the CSIRO data portal (https://doi.org/10.25919/5d39e494936c6), while the wild mouflons from the NextGen Project can be accessed via the project’s webpage in Ensembl (https://projects.ensembl.org/nextgen/). All mitogenomes were accessed via GeneBank and a list of accessions can be found in Supplementary Table 4. The dataset used for the demographic inference and *Momi2* models are available in Zenodo (https://doi.org/10.5281/zenodo.8017082). No new data was generated for this project. Supplementary Material is available at G3 online.
