# Supplementary material for: Demographic reconstruction of the Western sheep expansion from whole-genome sequences
Source: G3 (Bethesda). 2023 Sep 7;13(11):jkad199. doi: 10.1093/g3journal/jkad199 (PMC11648245; doi:10.1093/g3journal/jkad199)
Supplement: jkad199_Supplementary_Data [file jkad199_supplementary_data.zip › Supplemental_Figures_G3-2023-404492.pdf]

Demographic Reconstruction of the Western Sheep Expansion from Whole-Genome sequences

Pedro Morell Miranda, André ER Soares, Torsten Günther

Supplementary Material

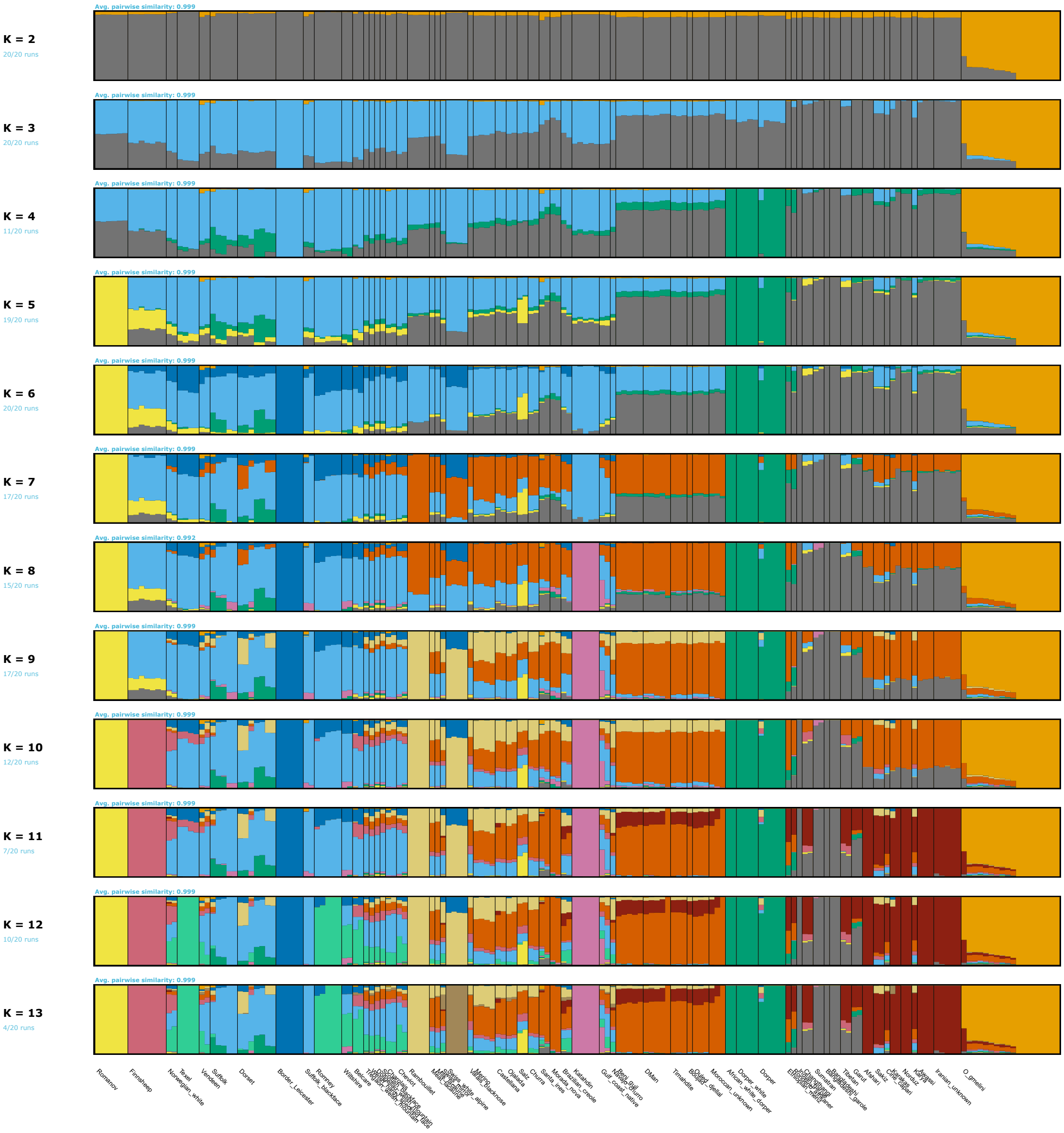

Figure S1: *ADMIXTURE* plots for $2 - 13Ks$.

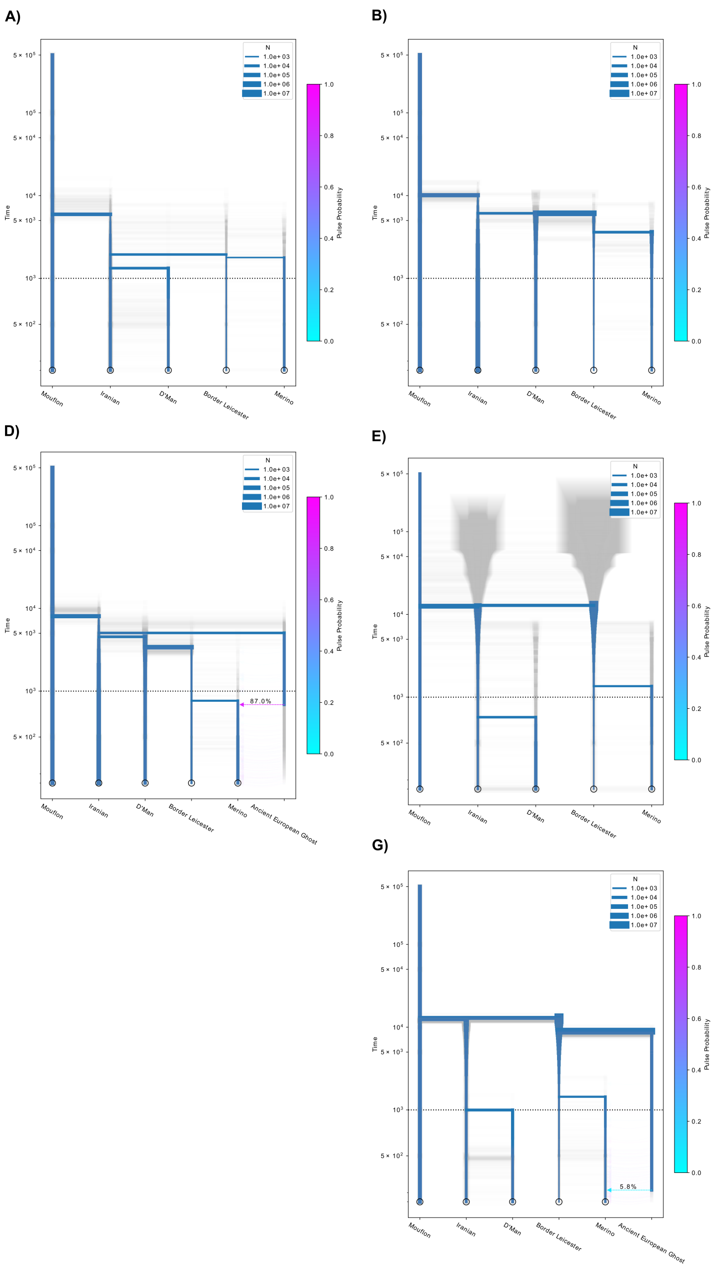

Figure S2: Results of *Momi2* demographic inference.

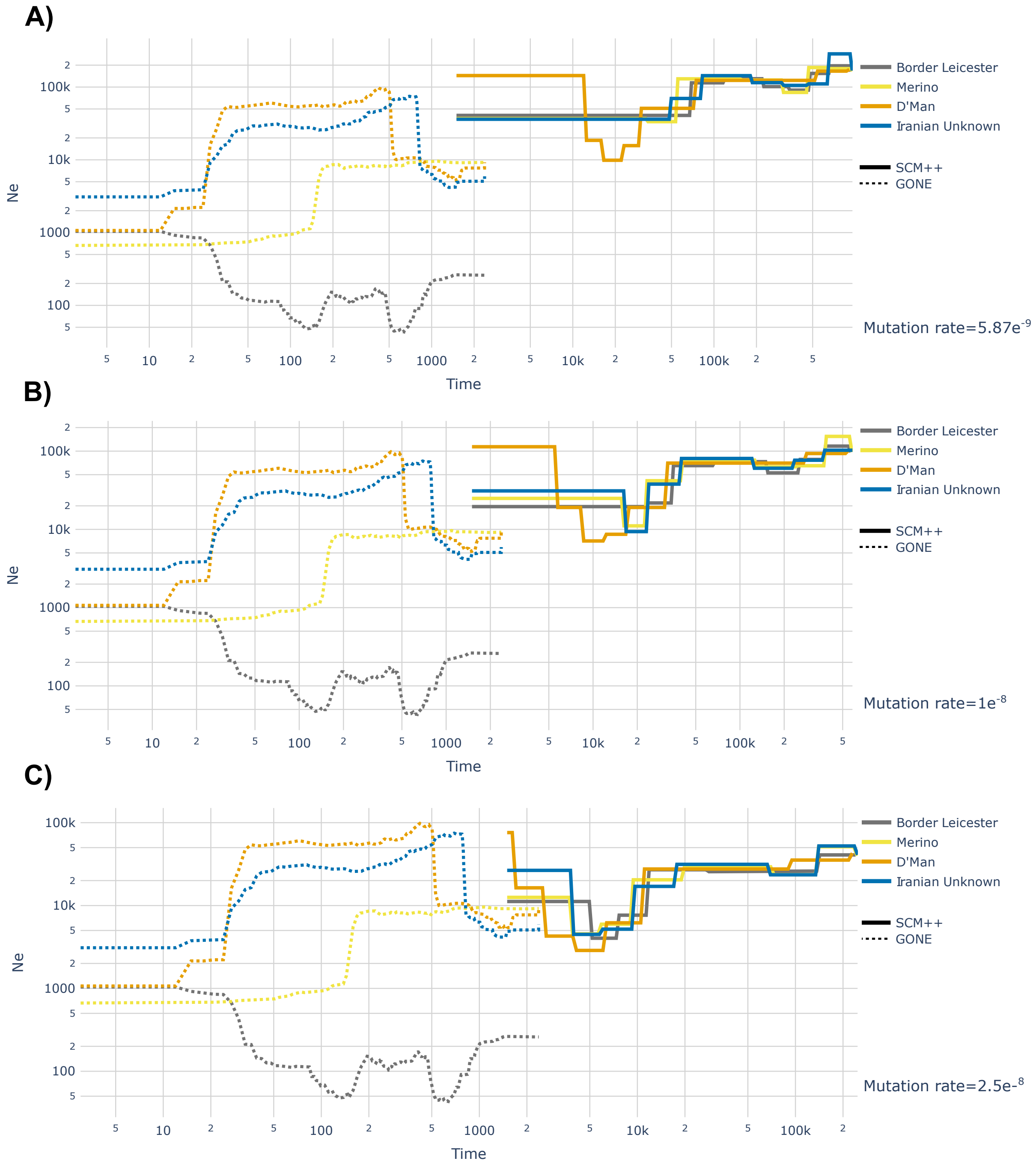

Figure S3: SMC++ and GONE results using 5.87×10^{-9}, 1×10^{-8} and 2.5×10^{-8} as mutation rates for SMC++.

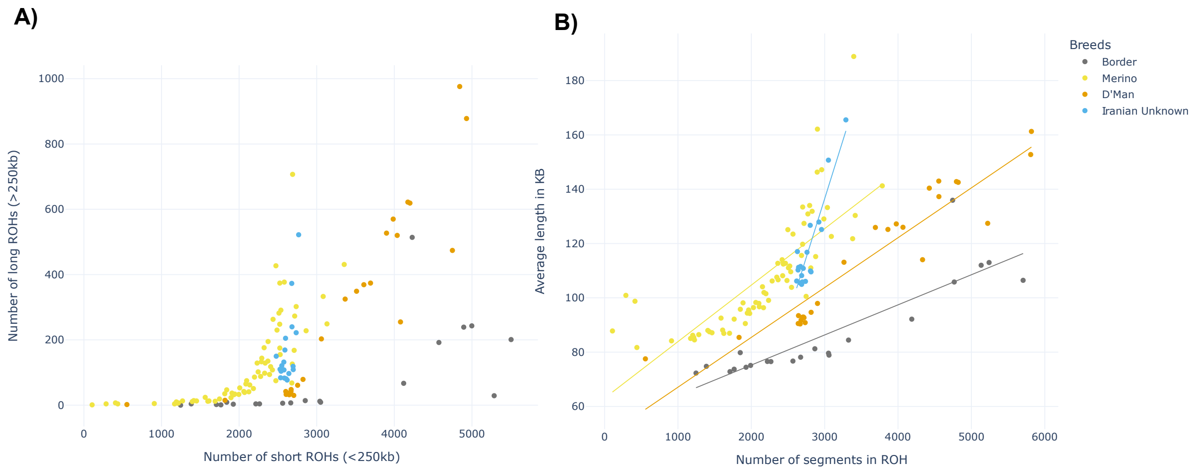

Figure S4: Comparison of the Runs of Homozygosity for the 4 domestic breeds. A) Number of long vs. short ROHs per sample and B) Average length of ROHs per sample vs. number of segments identified as ROHs

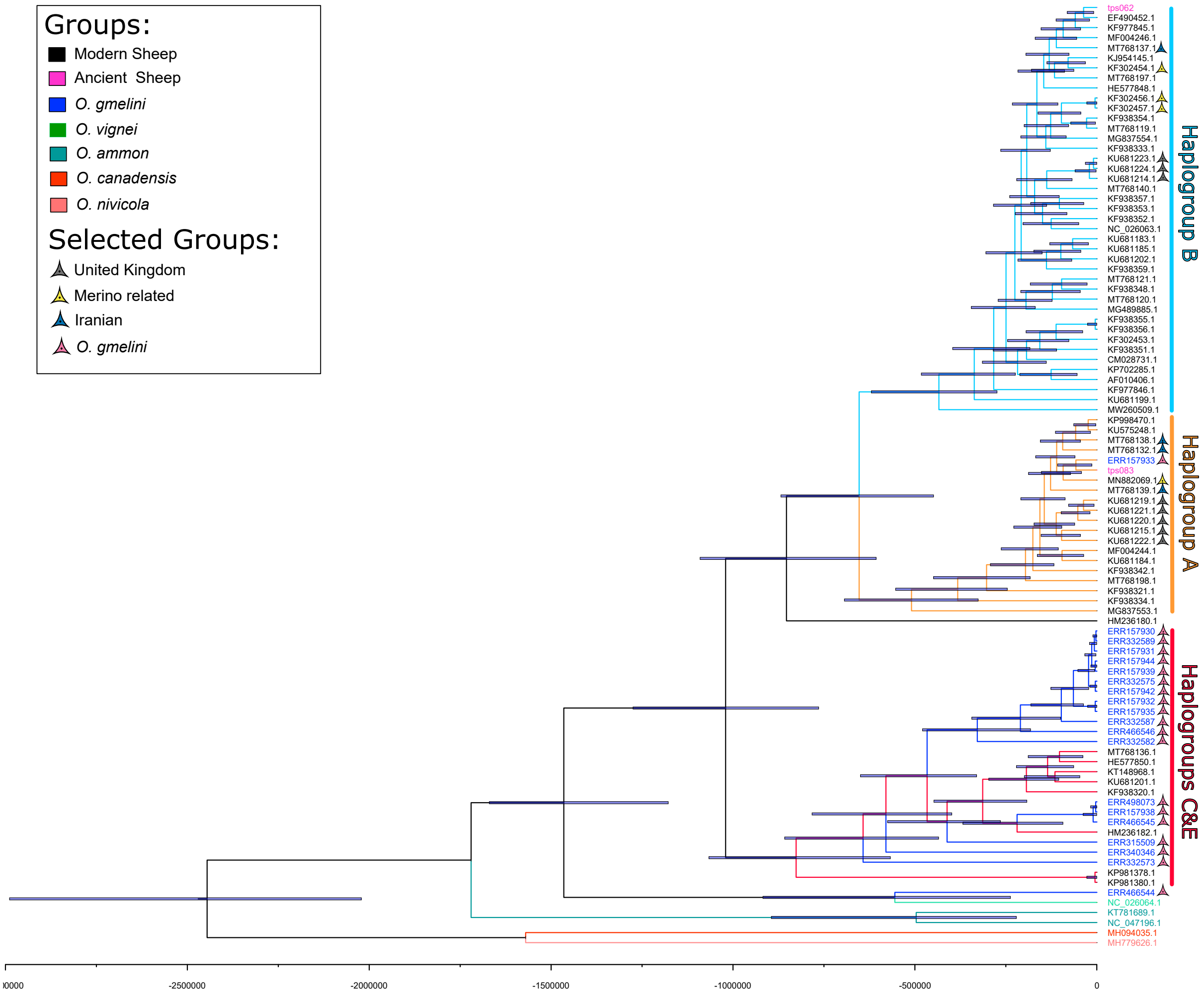

Figure S5: Bayesian Phylogenetic inference tree with *BEAST*. It shows domestic sheep clustering into three groups matching the mitochondrial haplogroups A, B and the CE complex. Split time estimation between the different mitochondrial sheep lineages and the results corroborate previous studies (Pedrosa et al. (2005); Meadows et al. (2011); Lv et al. (2015); Deng et al. (2020)), which found that the three major haplogroups (A, B and the CE cluster) significantly predate the domestication of this species usually assumed to have taken place around 10000 ya. Our estimates for the most recent common maternal ancestor between CE and (A, B) is 1020342 ya (95% HPD: 764505 to 1274862) while the split between haplogroups A and B was dated to 653310 ya (95% HPD: 448786 to 868374).

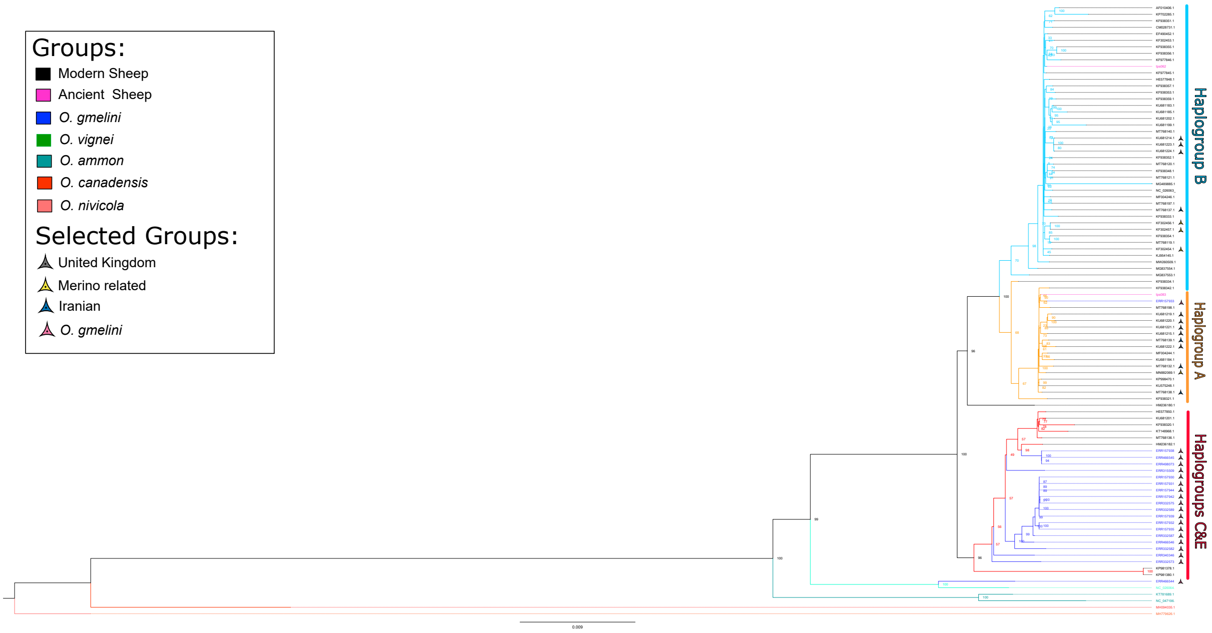

Figure S6: Mitochondrial Maximum Likelihood consensus tree showing similar structure to *BEAST*, splitting into three clusters matching with Haplogroups A, B and the CE complex. Within-haplogroup resolution, however, is lower, with several splits unresolved or with low support. Haplogroups A and B are ubiquitous across various Western sheep breeds making this analysis uninformative about the recent demographic history of the breeds discussed above. It does, however, provide additional insights into the relationship of the used mouflon populations to domestic sheep breeds: All but two Asiatic mouflons clustered within the CE complex. One mouflon falls within haplogroup A, close to the Neolithic *tps083*, while the last mouflon clusters with an urial (*O. vignei*).

References

J. Deng, X. L. Xie, D. F. Wang, C. Zhao, F. H. Lv, X. Li, J. Yang, J. L. Yu, M. Shen, L. Gao, J. Q. Yang, M. J. Liu, W. R. Li, Y. T. Wang, F. Wang, J. Q. Li, E. E. Hehua, Y. G. Liu, Z. Q. Shen, Y. L. Ren, G. J. Liu, Z. H. Chen, N. A. Gorkhali, H. E. Rushdi, H. Salehian-Dehkordi, A. Esmailizadeh, M. Nosrati, S. R. Paiva, A. R. Caetano, O. Štěpánek, I. Olsaker, C. Weimann, G. Erhardt, I. Curik, J. Kantanen, J. M. Mwacharo, O. Hanotte, M. W. Bruford, E. Ciani, K. Periasamy, M. Amills, J. A. Lenstra, J. L. Han, H. P. Zhang, L. Li, and M. H. Li. Paternal Origins and Migratory Episodes of Domestic Sheep. *Current Biology*, 30(20):4085–4095.e6, oct 2020. ISSN 18790445. doi: 10.1016/j.cub.2020.07.077.

F.-H. Lv, W.-F. Peng, J. Yang, Y.-X. Zhao, W.-R. Li, M.-J. Liu, Y.-H. Ma, Q.-J. Zhao, G.-L. Yang, F. Wang, J.-Q. Li, Y.-G. Liu, Z.-Q. Shen, S.-G. Zhao, E. Hehua, N. A. Gorkhali, S. M. Farhad Vahidi, M. Muladno, A. N. Naqvi, J. Tabell, T. Iso-Touru, M. W. Bruford, J. Kantanen, J.-L. Han, and M.-H. Li. Mitogenomic Meta-Analysis Identifies Two Phases of Migration in the History of Eastern Eurasian Sheep. *Molecular Biology and Evolution*, 32(10):2515–2533, oct 2015. ISSN 0737-4038. doi: 10.1093/MOLBEV/MSV139. URL `https://academic.oup.com/mbe/article/32/10/2515/1209894`.

J. R. Meadows, S. Hiendleder, and J. W. Kijas. Haplogroup relationships between domestic and wild sheep resolved using a mitogenome panel. *Heredity*, 106(4):700–706, apr 2011. ISSN 0018067X. doi: 10.1038/hdy.2010.122. URL `/pmc/articles/PMC3183909/?report=abstract` `https://www.ncbi.nlm.nih.gov/pmc/articles/PMC3183909/`.

S. Pedrosa, M. Uzun, J. J. Arranz, B. Gutiérrez-Gil, F. San Primitivo, and Y. Bayón. Evidence of three maternal lineages in near eastern sheep supporting multiple domestication events. *Proceedings of the Royal Society B: Biological Sciences*, 272(1577):2211–2217, oct 2005. ISSN 14712970. doi: 10.1098/rspb.2005.3204. URL `/pmc/articles/PMC1559946/?report=abstract` `https://www.ncbi.nlm.nih.gov/pmc/articles/PMC1559946/`.
